# Human papillomavirus infection: protocol for a randomised controlled trial of imiquimod cream (5%) versus podophyllotoxin cream (0.15%), in combination with quadrivalent human papillomavirus or control vaccination in the treatment and prevention of recurrence of anogenital warts (HIPvac trial)

**DOI:** 10.1186/s12874-018-0581-z

**Published:** 2018-11-06

**Authors:** Macey L. Murray, Jade Meadows, Caroline J. Doré, Andrew J. Copas, Lewis J. Haddow, Charles Lacey, Mark Jit, Kate Soldan, Kate Bennett, Michelle Tetlow, Mayura Nathan, Richard Gilson

**Affiliations:** 1 Comprehensive Clinical Trials Unit, Institute of Clinical Trials and Methodology, University College London, Gower Street, London, WC1E 6BT UK; 2 UCL Centre for Clinical Research in Infection and Sexual Health, The Mortimer Market Centre, Institute for Global Health, University College London, London, WC1E 6JB UK; 3 Centre for Immunology and Infection, Hull York Medical School, University of York, York, YO10 5DD UK; 4 Department of Infectious Disease Epidemiology, London School of Hygiene and Tropical Medicine, London, WC1E 7HT UK; 5 Public Health England, London, NW9 5EQ UK; 6 Homerton Anal Neoplasia Service, Homerton University Hospital NHS Foundation Trust, London, E9 6SR UK

**Keywords:** Podophyllotoxin, Imiquimod, Quadrivalent HPV vaccine, Human papillomavirus, Anogenital warts

## Abstract

**Background:**

Anogenital warts are the second most common sexually transmitted infection diagnosed in sexual health services in England. About 90% of genital warts are caused by human papillomavirus (HPV) types 6 or 11, and half of episodes diagnosed are recurrences. The best and most cost-effective treatment for patients with anogenital warts is unknown. The commonly used treatments are self-administered topical agents, podophyllotoxin (0.15% cream) or imiquimod (5% cream), or cryotherapy with liquid nitrogen. Quadrivalent HPV (qHPV) vaccination is effective in preventing infection, and disease, but whether it has any therapeutic effect is not known.

**Methods and design:**

To investigate the efficacy of clearance and prevention of recurrence of external anogenital warts by topical treatments, podophyllotoxin 0.15% cream or imiquimod 5% cream, in combination with a three-dose regimen of qHPV or control vaccination. 500 adult patients presenting with external anogenital warts with either a first or subsequent episode of anogenital warts will be entered into this randomised, controlled partially blinded 2 × 2 factorial trial.

**Discussion:**

The trial is expected to provide the first high-quality evidence of the comparative efficacy and cost-effectiveness of the two topical treatments in current use, as well as investigate the potential benefit of HPV vaccination, in the management of anogenital warts.

**Trial registration:**

The trial was registered prior to starting recruitment under the following reference numbers: International Standard Randomized Controlled Trial Number (ISRCTN) Registry - ISRCTN32729817 (registered 25 July 2014); European Union Clinical Trials Register (EudraCT) - 2013-002951-14 (registered 26 June 2013).

**Electronic supplementary material:**

The online version of this article (10.1186/s12874-018-0581-z) contains supplementary material, which is available to authorized users.

## Background

The most effective and cost-effective treatment for patients with anogenital warts is unknown. Genital warts are benign lesions which present as lumps or raised plaques in the skin of the anogenital area. Usually painless, but occasionally causing irritation or bleeding, they can be emotionally distressing, and often require prolonged, time consuming and uncomfortable treatment. Relapse after apparently successful treatment occurs frequently. Surgery may be required in persistent cases. About 90% of genital warts are caused by human papillomavirus (HPV) types 6 or 11, which are sexually transmitted.

There were 122,068 episodes of new or recurrent genital warts treated in sexual health services in England in 2016 [1], of which 49% were recurrent episodes. First episode genital warts accounted for 15% of new sexually transmitted infections (STIs) diagnosed, making it the second most common condition after chlamydia. The cost to the NHS of treating anogenital warts in 2016 is estimated at almost £14 million per year, of which about £6 million is for recurrent episodes [2].

Cryotherapy with liquid nitrogen is frequently used to treat anogenital warts, although this treatment requires equipment and facilities usually only available in hospital or specialist community settings, and appropriately trained staff. Effective treatment may be achieved with a single application, but more often requires repeated clinic attendance. Consequently, most cases of warts are now treated with self-administered topical agents, of which podophyllotoxin is the most common [3]. Podophyllotoxin has a chemotherapeutic action believed to be based on prevention of tubulin polymerisation required for microtubule assembly and inhibition of nucleoside transport through the cell membrane, leading to inhibition of growth of virally infected cells. It is available as a solution or a cream and has been studied in a number of randomised trials [3–11]. The cream (Warticon®, Glaxo SmithKline) includes the active agent at a lower concentration than the solution (0.15% versus 0.5%) but is generally considered to be easier to apply, better tolerated, and have similar efficacy. An alternative topical treatment is imiquimod, but this is more expensive, so usually reserved for second-line therapy. Imiquimod is available as a 5% cream (Aldara®, Meda). Some studies have suggested that imiquimod is associated with less recurrence as a result of its mode of action as an immune response modifier [12, 13]. It is a toll-like receptor 7 agonist, and stimulates tissue macrophages to release interferon-alpha and other cytokines which trigger a local cell-mediated response. Imiquimod has no direct antiviral activity. The treatment response may be slower than podophyllotoxin. It is licensed for a treatment duration of up to 16 weeks; most patients will have responded by 8 weeks. The efficacy has been investigated in a number of trials [11, 14–21].

The efficacies of podophyllotoxin and imiquimod as initial therapy for anogenital warts have never been compared in an adequately-powered trial [22]. The only randomised trial comparing the two topical agents was under-powered (*n* = 51) and did not report recurrence rates [11]. There were similar clearance rates (75% vs. 72%, 95% confidence interval 53–98% and 52–86% respectively). A systematic review of wart treatment undertaken for the European guidelines for the treatment of genital warts [22], suggested that podophyllotoxin has a similar rate of initial clearance (43–70% at 4 weeks, compared to 55–81% clearance at 16 weeks for imiquimod), but recurrence rates may be lower with imiquimod (6–26% at 6 months for imiquimod, compared to 6–55% at 8–12 weeks for podophyllotoxin). The wide variation between reported studies is also likely related to differences in study design, including the outcome measures and timing. The review found no evidence of any single therapy being superior overall, largely due to the lack of high-quality comparative studies, with those reported being heterogeneous in design, and with high loss to follow-up. Until this comparison is resolved in randomised studies of sufficient size and robust design it remains impossible to firmly recommend one treatment over the other. UK national guidelines recommend that the choice of first line therapy be based on patient preference and morphology and distribution of lesions, with a clinic treatment algorithm to guide treatment [23].

It is not known whether the clearance or recurrence rate of anogenital warts can be improved by vaccination against HPV 6 and 11, initiated at the same time as topical therapy. HPV vaccines are currently licensed to prevent HPV-associated anogenital warts and cancers; quadrivalent HPV vaccine (qHPV; Gardasil®, Merck Sharp & Dohme) protects against genotypes 6, 11, 16 and 18, and from September 2012 has been the vaccine used in the national vaccination programme in the UK for girls aged 12–13 years. The potential role of the vaccine as therapy or secondary prevention for anogenital warts (or indeed any HPV-associated disease) has yet to be determined. While no randomised controlled trial (RCT) evidence exists, evidence that vaccination against HPV may offer a therapeutic or secondary preventative strategy comes from several sources. Firstly, there are case reports that clearance of anogenital warts may be enhanced by qHPV vaccine [24, 25]. Secondly, patients with anogenital warts or genital intraepithelial neoplasia (cervical [CIN], vulval [VIN] or vaginal [VaIN]) are at risk of re-infection with the same or different HPV types, or relapse of existing infection [26, 27]. Thirdly, limited evidence from placebo-controlled vaccine trials appears to show that women who are HPV seropositive, but DNA-negative for at least one HPV type at trial entry were protected against subsequent disease related to the HPV type to which they were previously exposed [28]. Also, women with genital lesions treated surgically while in the vaccine trial were less likely to develop recurrent or progressive disease if they were in the vaccine arm of the trial [29]. Fourthly, preliminary evidence suggests that the qHPV vaccine may reduce recurrences of respiratory papillomatosis (RRP) in children [30], and of anal intraepithelial neoplasia (AIN) [31], both conditions caused by vaccine-type HPVs. Finally, vaccine antibody responses are much stronger than those induced by natural infection [32], so a strategy of priming or boosting anti-HPV 6/11 responses with qHPV vaccine could influence the persistence of HPV 6/11 infection and therefore the rate of disease recurrence.

Studies of the treatment cost and quality of life impact of genital warts, as well as economic analyses of vaccinating against warts have been conducted [2, 33, 34]. These studies have documented significant negative impacts on quality of life and substantial health care service costs. The cost of imiquimod remains higher than that of podophyllotoxin. If the effectiveness of imiquimod proves superior, then an economic analysis would allow an assessment of the maximum cost difference that would warrant its use as first line therapy. All available treatments have significant failure and recurrence rates. By maximising initial response rates and reducing recurrence rates using first-line self-administered treatment, a randomised controlled trial has the capacity to reduce this health and quality of life burden for patients and improve cost effectiveness, now and in the future. Vaccination would add to the cost of treatment of patients with anogenital warts. If efficacy is demonstrated, then an economic analysis could determine at what level the increased treatment costs would be justified by reduced future healthcare costs and improved quality of life related to persistent or recurrent disease.

The proposed trial will assess the comparative efficacy of the two main topical treatments in current use, podophyllotoxin 0.15% cream and imiquimod 5% cream, and will investigate the potential therapeutic benefit of the qHPV vaccine in the management of patients with anogenital warts. The trial will also evaluate the relative costs of the two topical treatments, as well as of the novel use of qHPV vaccination for both treatment and secondary prevention. The adoption of a pragmatic trial design with broad entry criteria for the comparison of the two topical therapies means that the results can be generalised to the large number of health care settings where anogenital warts are treated. The topical therapies assessed and the potential (within the protocol) to use supplementary cryotherapy are closely aligned with current clinical practice.

## Methods

### Study design

The study is a randomised, controlled partially blinded 2 × 2 factorial design trial for the treatment of anogenital warts, with an accompanying economic analysis. Participants are allocated in equal numbers to all four combinations of the two topical treatments and two vaccines (Table 1). Analysis of the primary outcome will be based on logistic regression.

**Table 1:** Interventions received according to the 2 × 2 factorial trial design. Randomisation is 1:1 between the two topical cream arms and 1:1 for qHPV and placebo

		Topical creams	
		*Imiquimod*	*Podophyllotoxin*
Vaccines	*qHPV vaccine*	Arm A *n* = 125 Imiquimod cream for 16 weeks; qHPV vaccine at months 0, 2 and 6	Arm B *n* = 125 Podophyllotoxin cream for 4 weeks; qHPV vaccine at months 0, 2 and 6
	*Saline, placebo control*	Arm C *n* = 125 Imiquimod cream for 16 weeks; placebo vaccine at months 0, 2 and 6	Arm D *n* = 125 Podophyllotoxin cream for 4 weeks; placebo vaccine at months 0, 2 and 6.

### Study setting

The trial is run at selected sexual health clinics in England and Wales (see Additional file 1: Participating sites); about 80% of cases of genital warts treated in the NHS are treated in sexual health clinics.

### Study population

Those eligible to participate are adults aged 18 or over presenting to participating sexual health clinics with external anogenital warts which, in the opinion of the investigator, could be appropriately treated with either self-administered imiquimod or podophyllotoxin cream. Patients with either first episode or repeat episode anogenital warts are eligible.

The main exclusion criteria are those who have had treatment for warts in the past 3 months, and those who have previous qHPV vaccine; previous bivalent HPV vaccine is not an exclusion. Other exclusion criteria relate to contraindications to any of the products, including previous intolerance, pregnancy, lactation, and a total wart area in excess of 4 cm^2^, which would require treatment under medical supervision. Patients requiring topical steroids applied to the affected area, or on systemic immunosuppressive agents were also excluded.

Initially, participants with known HIV infection were excluded but in December 2015 the entry criteria were modified to allow their inclusion. The change in criteria allows the inclusion of the majority of HIV-positive individuals, but still excludes those with more severe immunosuppression, who may be less likely to respond to topical treatment or to HPV vaccine. Current evidence is that those HIV-positive individuals stable on antiretroviral treatment with a CD4 count above 350, and those not on treatment with CD4 count above 500, mount good responses to vaccines, and have similar responses to treatment for some other chronic viral infections, such as hepatitis C [35–37]. It was concluded that HIV-positive individuals fulfilling these criteria should not be excluded. This would allow an estimated 80% of HIV-positive patients with warts to be eligible. It would also be of benefit to observe if the response to the trial interventions was comparable in those with and without HIV infection, although it is acknowledged that the power to detect any such effect would be limited. As a precaution, HIV status was added as a stratification variable.

### Interventions

#### Topical treatment (for up to 16 weeks)

##### Imiquimod

Participants randomised to imiquimod are asked to apply the 5% cream for three days of the week (every other day). The cream should be applied at the participant’s bed time and left on overnight. The cream should be washed off after 6–10 h.

##### Podophyllotoxin

Participants randomised to podophyllotoxin are instructed to apply the 0.15% cream to the lesions twice a day for three consecutive days followed by no treatment for 4 days, in weekly cycles. The licensed treatment duration is 4 weeks, but it is common practice to extend this period if there is a partial response to therapy.

#### Vaccine treatment

##### qHPV vaccine

Quadrivalent HPV vaccine, Gardasil – Sanofi Pasteur MSD, given according to the licenced schedule at 0, 2, and 6 months; vaccine volume 0.5 ml; containing alum adjuvant.

##### Placebo

0.5 ml normal saline injection as control.

Participants are randomised to one of 4 groups (Table 1):

A. imiquimod cream plus qHPV vaccine;
B. podophyllotoxin cream plus qHPV vaccine;
C. imiquimod cream plus saline placebo injection.
D. podophyllotoxin cream plus saline placebo injection.

Allocation to the groups is carried out using minimisation with a random element, with gender, previous occurrence of warts, and trial site as stratification factors. HIV status was added as a stratification factor when the entry criteria were changed. Participant identifiers and trial arm allocation are computer generated using a secure on-line service, which requires entry of limited participant characteristics.

Topical creams are dispensed in unblinded packaging and vaccine treatment is dispensed in blinded packaging using vaccine codes. The vaccinations are administered by trained members of the clinic staff, who are not part of the trial team involved in trial assessments.

### Screening and baseline assessment

After assessing eligibility and obtaining written informed consent, information on the following is collected: the date of first presentation; dates of previous episodes, and treatment of warts; history of sexually transmitted infections and co-morbidities; history of recent sexual contacts; concomitant medication; and quality of life questionnaire. Baseline assessment includes examination of the anogenital area and documentation of the position and approximate number of warts (in categories: 1–5, 6–10, 11–20, > 20). The maximum diameter of the largest wart is recorded, measured against a size gauge. A symptom-directed general physical examination is performed if appropriate. A blood sample (for serum) and a swab of the lesions (for HPV DNA) are taken and stored, for future methodological studies. Randomised treatments are prescribed or administered and participants are supplied with information on their use, risks and side-effects. Participants are offered safer sex advice, and access to other sexual and reproductive health services as per routine care. For women of child bearing potential a pregnancy test is performed. Simple diary cards are provided for the participant to use as a reminder of when the treatment should be applied, record its use, record if, and when, warts have cleared and record symptom scores related to the topical treatment.

### Follow-up assessments

Routine follow up is for 48 weeks in total, with visits at 4, 8, 16, 24 and 48 weeks (see Table 2). Further topical treatment is issued according to the randomisation/reassessment at weeks 4 and 8. Vaccine is administered at weeks 8 and 24.

**Table 2:** Baseline and follow up assessments and procedures

Visit number	1	2	3	4	5	6	Extra visits if warts recur
Week	0 (baseline)	4 (± 1 week)	8 (± 2 weeks)	16 (± 3 weeks)	24 (± 3 weeks)	48 (± 5 weeks)	
Give Participant Information Sheet and go through trial with participant	X						
Check eligibility, complete and sign Consent Form	X						
Randomisation	X						
Record wart treatment	X	X	X	X	X	X	X
Review and record concomitant medication	X	X	X	X	X	X	X
Examine and record approximate number and location of warts/the absence of warts	X	X	X	X	X	X	X
Symptom-directed general examination	X						
Urine pregnancy test (βhCG) (women of child bearing potential only^a^)	X	X^a^	X^a^	X^a^	X^a^	X^a^	X^a^
Quality of life questionnaire (EuroQoL EQ-5D-5 L)	X	X	X	X	X	X	X
Assessment of tolerability		X	X	X	X	X	
Assessment of Adverse Events and Pregnancy		X	X	X	X	X	X
Assessment of treatment response and need for additional/altered treatment		X	X	X	X	X	X
Lesion swab for HPV detection (all participants, samples to be archived)	X						X
Blood sample (all participants, serum sample to be archived)	X					X	
Supply trial wart treatment	X	X	X				
Supply/apply additional/alternative wart treatment if required and as permitted in the protocol		X	X	X	X	X	X (from week 4 onwards)
Vaccination	X		X		X		
Provide diary card for self-treatment and self-examination record	X	X	X	X	X		
Collect/review diary card		X	X	X	X	X	X
Completion/review of electronic trial documentation	X	X	X	X	X	X	X

^a^Pregnancy test to be completed, if the participant has not adhered to using effective contraception and is being prescribed any trial topical treatment. Effective contraception defined per HMA CTFG guidance (see Additional file 2: Effective contraception)

Presence of warts is determined on examination by a member of the trial clinical team at each study visit to confirm clearance/recurrence. Participants are asked to return to the clinic early if they notice a recurrence so that this can be documented (and the date of recurrence recorded) and further treatment offered according to standard of care. If warts recur within the first 16 weeks the participant is prescribed the treatment that they were randomised to at baseline.

Cryotherapy should only be administered from 4 weeks (visit 2) onwards if in the opinion of the investigator this is in the best interests of the patient and after assessment of the response to topical treatment to date. Careful consideration should be given to delaying cryotherapy beyond 4 weeks where a response to topical treatment is observed in the interest of preserving the integrity of the randomised comparison. If a participant is unable to tolerate the allocated treatment, after dose modifications as appropriate, alternative treatment can be administered at the discretion of the investigator. For the purposes of the trial, early use (within 4 weeks of the start of randomised treatment) of alternative treatments and topical treatment switch to the opposite arm before 16 weeks is considered a topical treatment failure. Participants should continue to be followed up and receive vaccinations as per protocol.

After week 16, topical treatment can be changed at the discretion of the investigator, including a switch to the other randomised topical treatment. Timing of eventual wart clearance is recorded for the secondary outcome (time to clearance).

Participants are provided with a diary card to record treatments applied and the date the warts are last seen and/or recur, and if there are additional visits between week 16 and 48 for clinical care, a record of presence/absence of warts is made.

Routine visits include an assessment of treatment response, as reported by the participant, and observed by the clinician, adherence to the treatment regimen, tolerability, health-related quality of life, and the need for additional or extended treatment. Participants are also asked about work days lost due to clinic visits. Diary cards are collected. An end of trial blood sample is collected at week 48, and a lesion swab, to be stored for later HPV DNA detection is collected in the event of wart recurrence. Additional visits are arranged in the event of recurrences or other indications for additional treatment or review in line with routine clinical practice, or if significant adverse events occur that require medical assessment. In order to reduce the loss to follow-up rate, a small financial incentive is provided to those participants who attend the week 16 and week 48 visits.

### Primary outcome

A composite endpoint of wart clearance 16 weeks after starting treatment and remaining wart-free between 16 and 48 weeks. This captures both the initial clearance efficacy as well as the impact on relapse or recurrence.

### Secondary outcomes

The two components of the composite primary endpoint are considered as factor specific clinically important secondary outcomes:

- for topical treatment– proportion wart-free at 16 weeks.
- for vaccination– proportion remaining wart free between week 16 and week 48 in those with wart clearance at 16 weeks.

Other secondary outcomes are:

1. Proportion wart-free at the end of the assigned treatment course (4 or 16 weeks)
2. Proportion wart-free at the end of the assigned treatment course (4 or 16 weeks) without receiving additional treatment
3. Quantity of additional treatment (number of cryotherapy applications, additional weeks of podophyllotoxin or imiquimod) required to achieve clearance by 16 weeks
4. Proportion wart-free at 16 weeks without receiving additional treatment
5. Proportion experiencing complete wart clearance
6. Proportion experiencing wart recurrence/relapse after complete wart clearance
7. Time to complete wart clearance
8. Time from complete wart clearance to recurrence/relapse
9. Adverse events
10. Health-related quality of life, as measured by the Area Under the Curve for EQ-5D-5 L
11. Symptom scores
12. Total costs of treatment including prescribed agents and clinic visits

### Statistical considerations

#### Sample size

The trial was originally designed with a sample size of 1000 participants with equal numbers randomised to each of the two topical cream arms and each of the two vaccine groups in a 2 × 2 factorial design, so that allowing for 20% loss to follow-up 800 participants would contribute primary outcome data. The anticipated proportion achieving the primary endpoint in the less favourable topical treatment group was 35%, assuming a wart clearance rate of 50% within 16 weeks and a 30% subsequent recurrence rate. This sample size provided 80% power (at the 5% significance level) to detect an increase to 45% with the better treatment. It also provided 80% power to detect an increase from 35 to 45% in the primary endpoint from vaccination, as would arise if vaccination reduces the recurrence rate from 30 to 10% whilst leaving the wart clearance rate unchanged at 50%.

Owing to a lack of feasibility to achieve the proposed recruitment target of 1000, a revised sample size of 500 participants was proposed in February 2016. With 15% loss to follow-up, this would provide 52% power (at the 5% significance level) to detect the pre-specified difference in the combined primary endpoint.

It was expected that the main effect of the topical treatment would be on wart clearance, and the main effect of vaccination would be on wart recurrence. The power of the study to detect a clinically important difference in each of these secondary outcomes was therefore calculated in proposing the reduced trial size.

The revised sample size will provide 80% power at the 5% significance level, assuming 15% loss to follow up, to evaluate each of the two components of the primary outcome: 1) Proportion wart-free at 16 weeks; 2) Proportion remaining wart-free at 48 weeks among participants who are wart-free at 16 weeks. For the 16 week topical treatment outcome, a difference of 14% between topical arms (57% versus 43%) could be detected; for the 48 week vaccine outcome, a difference of 16% between vaccine arms (12% versus 28%) could be detected. These differences are considered to be clinically important and may influence management guidelines. A 5% significance level has been used for both calculations as there is a different outcome for each of the two factors to answer two independent questions.

#### Statistical methods

Analysis will be by intention-to-treat (where participants are analysed according to the treatment arm to which they were randomised), and will include all participants for whom an outcome is obtained. The primary analysis for both factors (podophyllotoxin vs. imiquimod, and qHPV vs. placebo vaccine) will be based on comparisons at the margins of the 2 × 2 table (Table 1), meaning all participants randomised to podophyllotoxin will be compared with all participants randomised to imiquimod, and all participants randomised to qHPV vaccine will be compared with all participants randomised to saline placebo.

We do not anticipate a substantial interaction between topical treatment and vaccination. However, as a secondary analysis, we will perform an interaction test between the two factors, and present results from a four-arm analysis (where each of the four treatment groups is regarded as a separate treatment arm), as is recommended for factorial trials [38, 39].

The primary outcome (the composite endpoint of wart clearance 16 weeks after starting treatment and remaining wart-free between 16 and 48 weeks) and its two components (wart clearance at 16 weeks, and no wart recurrence by 48 weeks in those with wart clearance at 16 weeks) will be analysed using a logistic regression model, and will be adjusted for gender, previous occurrence of warts, HIV status and site as stratification factors [40, 41], and will include both treatment factors (topical treatment and vaccination) as covariates. Treatment effect estimates, 95% confidence intervals, and two-sided *p*-values will be reported for each outcome measure. Multiple imputation using chained equations [42] will be used to impute data for missing follow-up visits; the imputation model and number of imputations will be fully specified in the statistical analysis plan. Participants who do not attend any follow-up visits will be excluded from the analysis. A detailed statistical analysis plan will be written prior to final analysis and will include the planned analyses for the primary outcome and all secondary outcomes, subgroup analyses and sensitivity analyses.

#### Economic evaluations

Economic outcomes will be collected in the same way as previous studies by the investigators [33]. Health-related quality of life will be assessed using the EuroQol EQ-5D-5 L. Information on types of treatments given and number of visits made will be collected. These will be combined with unit costs from standard NHS sources as well as costs and treatment patterns from a previous economic study [33] to evaluate treatment costs. As the previous study was conducted in 2009–10, a panel of clinicians will be consulted to see if typical treatment patterns have changed since that time. Clinical, quality of life and cost data will be used to assess the relative cost-effectiveness of alternative treatment options, with or without vaccination. Cost-effectiveness analyses will be conducted according to the reference case used by the National Institute for Health and Care Excellence to ensure comparability with other economic evaluations.

## Discussion

Anogenital warts are the second most common sexually transmitted infection seen in patients presenting to sexual health services in the UK. Although benign, they can cause troublesome symptoms and distress. Treatment can be slow, time-consuming and uncomfortable, and recurrence is common. Since 2012, the HPV vaccine programme in 12–13 year-old girls has used the quadrivalent vaccine which is effective against HPV 6 and 11, in addition to the high-risk HPV16/18 types which cause most genital cancer. While the incidence of anogenital warts in young adults is beginning to fall, the current programme is not predicted to result in elimination and effective treatment for warts will continue to be required. The best first line topical treatment for anogenital warts has not been established in clinical trials, so that treatment guidelines lack a firm basis for their recommendations. The HPV vaccine is indicated to prevent infection; a therapeutic effect, although suggested, has not been established in a clinical trial.

The trial design is pragmatic, intended to allow as wide a range of participants as possible, to enable the results to be generalised. Both patients with a first presentation of warts, and those with a previous history are included. Those with warts that have been treated recently (within the last 3 months) are excluded, on the grounds that we are not investigating the efficacy in warts resistant to standard treatment.

The study does not have a placebo topical treatment arm to be able to detect an effect of vaccine on clearance in the absence of any topical treatment. While of scientific interest, this would have increased the size of the study, and make it harder to recruit given the current standard of care.

No change of topical treatment before 16 weeks is allowed as this is the primary endpoint for the topical treatment effect, even though the licensed duration of treatment differs for the two products. The addition of cryotherapy after week 4 is permitted because of current practice in the UK. A failure of response after 4 weeks of podophyllotoxin, which is the licensed duration of treatment, would currently prompt consideration of alternative treatment. In order to retain participants in the study, continuation of podophyllotoxin up to 16 weeks is therefore permitted within the protocol. Use of cryotherapy after 4 weeks is at the discretion of the investigator; cryotherapy use is recorded. A cryotherapy-alone arm was not included as being beyond the remit of this study, and because of the implications for the size of the trial.

Two limitations arose during the implementation of the study. It was intended that the vaccine would be blinded, with matching syringes containing a saline placebo. However, the vaccine is supplied in a bespoke pre-filled syringe and we were unable to source un-filled syringes to make a matching placebo. In order to minimise the risk of un-blinding, the vaccine is supplied in an opaque, sealed pouch contained within an outer carton. The vaccine or placebo is administered by a health care worker who is not involved in participant recruitment or follow up assessments, under instructions to avoid showing the vaccine syringe to either the participant or the trial team.

The study was designed with a sample size of 1000 participants. However, identifying trial sites in the UK with the resources and infrastructure to recruit participants proved to be very difficult. In order to complete the trial within the agreed timelines set by the funder, even with a no-cost extension, it was agreed that a reduced sample size would have to be accepted. The trial will therefore be underpowered for the combined primary endpoint. However, the power will be better for the two most important secondary endpoints, the rate of clearance at week 16 and the rate of recurrence at week 48, in those who have cleared warts by week 16.

The trial will provide the first high-quality evidence of the comparative efficacy of the two main topical treatments in current use, as well as the first randomised trial to investigate the potential therapeutic benefit of an HPV vaccine in the management of patients with anogenital warts.

## Additional files

Additional file 1: Participating sites. (DOCX 14 kb)

Additional file 2: Effective contraception. (DOCX 27 kb)

## Abbreviations

AIN: Anal intraepithelial neoplasia
CIN: Cervical intraepithelial neoplasia
HIV: Human immunodeficiency virus
HPV: Human papillomavirus
qHPV: Quadrivalent human papillomavirus
RCT: Randomised controlled trial
RRP: Recurrent respiratory papillomatosis
STI: Sexually transmitted infection
VaIN: Vaginal intraepithelial neoplasia
VIN: Vulval intraepithelial neoplasia
