# Supplementary material for: Human papillomavirus infection: protocol for a randomised controlled trial of imiquimod cream (5%) versus podophyllotoxin cream (0.15%), in combination with quadrivalent human papillomavirus or control vaccination in the treatment and prevention of recurrence of anogenital warts (HIPvac trial)
Source: BMC Med Res Methodol. 2018 Nov 6;18:125. doi: 10.1186/s12874-018-0581-z (PMC6220496; doi:10.1186/s12874-018-0581-z)
Supplement: Supplementary file 1 — Participating sites. (DOCX 14 kb) [file 12874_2018_581_MOESM1_ESM.docx]

# **Additional file 1: Participating sites**

| **Site name** | **NHS Trust** | **Principal Investigator** |
| --- | --- | --- |
| Mortimer Market Centre | Central and North West London NHS Foundation Trust | Dr Richard Gilson |
| YorClinic | York Hospital NHS Foundation Trust | Prof Charles Lacey |
| Homerton University Hospital | Homerton University Hospital NHS Foundation Trust | Dr Mayura Nathan |
| Royal Sussex County Hospital | Brighton and Sussex University Hospitals NHS Trust | Dr Daniel Richardson |
| Birmingham Heartlands Hospital | Heart of England NHS Foundation Trust | Dr David White |
| Manchester Centre for Sexual Health | Central Manchester University Hospitals NHS Foundation Trust | Dr Gabriel Schembri |
| Southend University Hospital | Southend University Hospital NHS Foundation Trust | Dr Mohd Abu Bakar |
| Royal Liverpool Hospital | Royal Liverpool and Broadgreen University Hospitals NHS Trust | Dr Mark Lawton |
| Royal Bournemouth Hospital | The Royal Bournemouth and Christchurch Hospitals NHS Foundation Trust | Dr Elbushra Herieka |
| James Cook University Hospital | South Tees Hospitals NHS Foundation Trust | Dr David Chadwick |
| Medway Maritime Hospital | Medway NHS Foundation Trust | Dr Rajesh Hembrom |
| Courtyard Clinic, St George's University Hospital | St George's University Hospitals NHS Foundation Trust | Dr Phillip Hay |
| Royal Hallamshire Hospital | Sheffield Teaching Hospitals NHS Foundation Trust | Dr Karen Rogstad |
| Trafalgar Clinic, Queen Elizabeth Hospital | Lewisham and Greenwich NHS Trust | Dr Stephen Kegg |
| Caldecot Centre, King's College Hospital | King's College Hospital NHS Foundation Trust | Dr Mannampallil Itty Samuel |
| New Croft Centre, Newcastle upon Tyne | The Newcastle upon Tyne Hospitals NHS Foundation Trust | Dr Mayur Chauhan |
| Cardiff Royal Infirmary | Cardiff and Vale University Health Board | Dr Laura Cunningham |
| The Gate Clinic, Kent Community Health | Kent Community Health NHS Foundation Trust | Dr Anitha Vidhyadharan |
| The Park Centre for Sexual Health,  Weymouth Community Hospital | Dorset County Hospital NHS Foundation Trust | Dr Cecilia Priestley |
| London Road Community Hospital | Derby Teaching Hospitals NHS Foundation Trust | Dr Ade Apoola |
| St Mary's Hospital, Paddington | Imperial College Healthcare NHS Trust | Dr Angela Bailey |
| Hope House, Gloucestershire Royal Hospital | Gloucestershire Care Services NHS Trust | Dr Andrew de Burgh-Thomas |
