# Supplementary material for: Human papillomavirus infection: protocol for a randomised controlled trial of imiquimod cream (5%) versus podophyllotoxin cream (0.15%), in combination with quadrivalent human papillomavirus or control vaccination in the treatment and prevention of recurrence of anogenital warts (HIPvac trial)
Source: BMC Med Res Methodol. 2018 Nov 6;18:125. doi: 10.1186/s12874-018-0581-z (PMC6220496; doi:10.1186/s12874-018-0581-z)
Supplement: Supplementary file 2 — Effective contraception. (DOCX 27 kb) [file 12874_2018_581_MOESM2_ESM.docx]

# Additional file 2: Effective contraception

1. Established use of oral, injected or implanted hormonal methods of contraception.
2. Placement of an intrauterine device (IUD) or intrauterine system (IUS).
3. Female condom or occlusive cap (diaphragm or cervical/vault caps) with spermicidal foam/gel/film/cream/suppository.
4. Regular and consistent use of male condoms.
5. Male sterilisation (with the appropriate post-vasectomy documentation of the absence of sperm in the ejaculate).
6. True abstinence: When this is in line with the preferred and usual lifestyle of the subject.

These are according to Heads of Medicines Agencies Clinical Trial Facilitation Group (HMA CTFG) 2014 guidance on contraception and pregnancy testing in clinical trials.

(<http://www.hma.eu/fileadmin/dateien/Human_Medicines/01-About_HMA/Working_Groups/CTFG/2014_09_HMA_CTFG_Contraception.pdf>.)
